# Paraben exposure through drugs in the neonatal intensive care unit: a regional cohort study

**DOI:** 10.3389/fphar.2023.1200521

**Published:** 2023-06-08

**Authors:** Silvia Iacobelli, Marie Commins, Simon Lorrain, Beatrice Gouyon, Duksha Ramful, Magali Richard, Anthony Grondin, Jean-Bernard Gouyon, Francesco Bonsante

**Affiliations:** ^1^ Néonatologie, Réanimation Néonatale et Pédiatrique, CHU La Réunion, Site Sud, Saint Pierre, France; ^2^ Centre d’Études Périnatales de l’Océan Indien, Université de la Réunion, Saint-Pierre, France; ^3^ Service de Réanimation Néonatale, CHU La Réunion, Saint-Denis, France

**Keywords:** neonatal intensive care unit, computerized prescriber order entry, drug excipients, endocrine disruptors, data collection, EFSA European Food Safety Authority, 1000-day window, extremely low birth weight (ELBW) infant

## Abstract

**Background and objectives:** Environmental factors influence the development of very preterm infants (VPIs, born at less than 32 weeks of gestation). It is important to identify all potential sources of paraben exposure in these vulnerable infants. We aimed to quantify paraben exposure via drug administration in a cohort of VPI cared for in neonatal intensive care units (NICUs).

**Methods:** A prospective, observational study was carried out over a five-year period in a regional setting (two NICUs using the same computerized order-entry system). The main outcome was exposure to paraben-containing drugs. The secondary outcomes were: time of the first exposure, daily intake, number of infants exceeding paraben acceptable daily intake (ADI: 0–10 mg/kg/d), duration of exposure, and cumulative dose.

**Results:** The cohort consisted of 1,315 VPIs [BW 1129.9 (±360.4) g]. Among them, 85.5% were exposed to paraben-containing drugs. In 40.4% of infants, the first exposure occurred during the second week of life. Mean paraben intake and duration of exposure were, respectively, 2.2 (±1.4) mg/kg/d and 33.1 (±22.3) days. The cumulative paraben intake was 80.3 (±84.6) mg/kg. The ADI was exceeded in 3.5% of exposed infants. Lower GA was associated with higher intake and longer exposure (*p* < 0.0001). The main molecules involved in paraben exposure were: sodium iron feredetate, paracetamol, furosemide, and sodium bicarbonate + sodium alginate.

**Conclusion:** Commonly used drugs are potential source of parabens, and ADI can be easily exceeded in VPIs cared for in NICUs. Efforts are needed to identify paraben-free alternative formulations for these vulnerable infants.

## Introduction

Parabens, alkyl esters of p-hydroxybenzoic acid, are chemical compounds widely used as preservatives in cosmetics, food and beverages, and industrial and pharmaceutical products. The use of methylparaben (MP), ethylparaben (EP), propylparaben (PP), and butylparaben (BP) is common, as an additive, in oral and parenteral pharmaceutical formulations, due to their antibacterial activity. Since the publication in 2004 of the first measurement of parabens in human breast cancer tissues (Darbre et al., 2004), numerous *in vitro* and *in vivo* studies have proven the estrogenic effects of parabens, together with their antiandrogenic properties and genotoxic activity (Darbre and Harvey, 2008). More recently, cellular and animal studies highlighted the adverse health risks of these “endocrine disruptors,” especially following perinatal exposure. In a female rat model, the postnatal exposure to MP resulted in a significant delay in the date of vaginal opening and in a decreased number of corpora lutea (Vo et al., 2010). In male rats, perinatal exposure to BP induced a modification in the process of spermatogenesis (Zhang et al., 2014). It has also been demonstrated that exposure of murine cells to parabens promotes lipid accumulation and adipogenesis and may contribute to obesity in later life (Hu et al., 2013).

According to the hypothesis of the “Developmental Origins of Health and Disease” (DOHaD) (Barker, 2007), nutrient and environmental agent exposures (including endocrine disruptors) during pregnancy may affect fetal and/or newborn development, resulting in offspring occurrence of “metabolic syndrome” (chronic non-communicable diseases [CNCD]: obesity, type 2 diabetes, and cardiovascular diseases).

Very preterm infants (VPIs, born at less than 32 weeks of gestation) are newborn babies at an increased risk of “metabolic syndrome” throughout multiple mechanisms (Markopoulou et al., 2019). First, VPIs have a low birth weight (birth weight less than 2,500 g). Second, exposure to infection/inflammation (often involved in premature birth) and therapeutic or nutritional strategies required for the management of these infants, are additional risk factors for the later onset of CNCD. Finally, the effects of endocrine disruptors can be particularly concerning in VPIs, as hormonal disruptions can cause permanent alterations in hormonal signaling pathways and developmental processes during the early life of vulnerable offspring, according to animal and human studies (Nelson et al., 2020). However, the exact association between paraben exposure during perinatal life and the prevalence of specific adverse health outcomes is not fully established yet in human epidemiological studies (Philippat et al., 2014; Jamal et al., 2019; Philippat et al., 2019). Such results can be explained by the complexity of the developmental effects of the environment on future health.

Moreover, the dose–response relationship between paraben exposure and increased risk of adverse effects is not defined, as toxicological information on the acceptable human exposure dose to parabens is uncertain and inaccurate. In fact, non-observed-adverse-effects-levels (NOAELs) for both MP and EP have been identified in long-term toxicity studies and in studies of no effect on sex hormones and male reproductive organs in exposed juvenile rats (Oishi, 2004). Based on these findings, the European Food Safety Authority (EFSA) set forth the acceptable daily intake (ADI) of 0–10 mg/kg/d for the sum of MP and EP (EFSA, 2004). This amount is until now indicated as the ADI for parabens by the European Medicines Agency (European Medicines Agency, 2015). Expert panel reviews (No, 2008) and *in vitro* toxicological tests (Svobodova et al., 2023) confirmed the safety of use of some allowed parabens at the highest recommended concentrations in cosmetic products and drugs.

It is difficult to extrapolate these results to VPIs. First, the margin of safety for infants ranged from 6,000 for single paraben exposure to 3,000 for multiple paraben exposure *vs.* exposure limits from 1,690 to 840 for adults (No, 2008). Moreover, since exposure to parabens is ubiquitous in these vulnerable infants, there is a need to identify sources, quantify them in terms of dose and duration, and mitigate exposure. VPIs are cared for in neonatal intensive care units (NICUs), where they are often in intimate contact with medical appliances containing parabens and other endocrine disruptors (Iribarne-Durán et al., 2019). Among neonates, VPIs are the most exposed to drugs during their hospital stay in the NICU (Gouyon et al., 2019). These infants also have a risk of accumulation of drugs and drug-excipients, due to their renal immaturity (Iacobelli and Guignard, 2021).

The main objective of this research was to quantify the exposure to parabens via drug administration in a regional cohort of preterm infants born at less than 32 weeks of gestation.

The secondary objectives were to measure the daily intake, the duration, and the cumulative dose of paraben intake and to analyze the evolution of exposure rates over time.

## Methods

### Study design and setting

The study was a prospective observational cohort study conducted in the two tertiary-care NICUs of Reunion Island University Hospital (France) from 1 January 2017 to 31 December 2021.

### Study population and inclusion criteria

All infants born between 24 and 31 weeks of gestation, and admitted for their first hospitalization in one of the two NICUs, were eligible.

This is a regional-based study, as only two tertiary-care NICUs exist in Reunion Island, a French overseas department in the Indian Ocean, and they provide care to all VPIs born in the region.

### Data sources

For the medical prescription, the two NICUs utilize the same computerized prescriber order entry (CPOE) system, with a clinical decision support system (Logipren^®^).

The CPOE (Logipren^®^) has been described in previous studies (Gouyon et al., 2017; Gouyon et al., 2019). This CPOE allows medication prescription according to gestational age (GA), postnatal age, post-conceptional age, body weight at the day of the prescription, and indication. The system provides a complete preselected drug prescription with drug dose, modality of administration, preparation modalities, and warnings. The prescriber has to indicate the daily body weight, choose the drug, specify its indication, and modify or confirm the prescription after a warning. At the time of prescription, all electronic prescriptions are prospectively and automatically stored on local computer servers. After full anonymization (deidentification) within each unit, they are sent to the same data warehouse for subsequent analysis.

Data on parabens contained in drugs were collected from the Thériaque^®^ database (Thériaque, 2008). This prescription products’ guide provides information on all medications available in France and specifies pharmaceutical form, composition, recommendations, indications, and precautions for use. In the composition section, it is possible to see whether the medication contain parabens, as well as the paraben concentration [MP, EP, PP, and BP (mg/mL)] in each formulation. We searched for all the 250 drugs available in the CPOE using the Thériaque^®^ database, and we identified 15 medications containing parabens (Table 1).

**TABLE 1:** Drugs containing parabens and administered to the cohort during the study period.

INN	Trend name and pharmaceutical form	MP	EP	PP	BP	Total
Acetylcysteine*	FLUIMUCIL^®^ [5 g/25 mL], injection	1	0	0	0	1
Alfacalcidol	UN-ALFA 0,10 µG oral drops, bottle 10 mL	1.5	0	0	0	1.5
Amphotericin B	FUNGIZONE^®^ 10%, suspension	1.15	0	0.35	0	1.5
Sodium alginate + sodium bicarbonate	GAVISCON^®^, suspension 10 mL	4	0	0.6	0	4.6
Biotin*	BIOTINE^®^ [5 mg/1 mL], injection	0.8	0	0.1	0	0.9
Domperidone	MOTILIUM^®^ [1 mg/mL], suspension, bottle 200 mL	1.8	0	0.2	0	2
Sodium feredetate	FERROSTRANE^®^ 0.68% solution	1	0	0.2	0	1.2
Furosemide	LASILIX^®^ [10 mg/mL], solution	1	0	0.5	0	1.5
Josamycin	JOSACINE^®^ [250 mg/5 mL], suspension	1.325	0	0.175	0	1.5
Lamivudine	EPIVIR^®^ [10 mg/mL], solution	ND	0	ND	0	ND
Levetiracetam	KEPPRA^®^ [100 mg/mL], solution, bottle 150 mL	2.7	0	0.3	0	3
Metronidazole	FLAGYL^®^ [125 mg/5 mL] solution	0.8	0	0.2	0	1
Nystatin	MYCOSTATINE^®^ [100,000 UI/mL] suspension	1	0	0.2	0	1.2
Paracetamol	DOLIPRANE^®^ 2.4%, suspension	ND	ND	ND	0	1.5
Rifampicin	RIFADINE^®^ 2%, suspension, bottle 120 mL	ND	0	ND	0	ND
Sulfamethoxazole + trimethoprim	BACTRIM^®^, suspension, bottle 100 mL	0.5	0	0.1	0	0.6

All paraben concentrations are expressed as mg/mL. All pharmaceutical forms were for oral route administration, unless indicated by*. INN, International Non-proprietary Name; MP, methylparaben; EP, ethylparaben; PP, propylparaben; BP, butylparaben; ND, amount of parabens not defined in the Thériaque^®^ database.

### Outcomes

The main outcome of interest was exposure to drugs containing parabens. Exposure was defined as having at least one dose of any drug administered at any time during the hospital stay, containing a paraben. No exposure was defined if there were no documented doses administered during that time.

The secondary outcomes were the following: time of the first exposure to drugs containing parabens, expressed as day of life (DOL) of the first administration; daily paraben intake (mg/kg/d); number of infants receiving paraben intake >10 mg/kg/d; number of days with paraben intake >10 mg/kg/d; duration of exposure, classified by total days of exposure; and cumulative dose (mg/kg) of parabens during the hospital stay. We assessed the evolution of exposure rates to drugs containing parabens over the study period.

### Statistical analysis

Categorical variables were presented as numbers and proportions. Continuous variables were presented as the mean ± standard deviation or median and range. Normality was checked using the Shapiro–Wilk test. The Cochran–Armitage test for trend was applied. Bivariate comparisons were performed using χ2 test or Student’s t-test for qualitative variables. A *p*-value less than 0.05 was considered significant. Statistical analysis was conducted using SAS^®^ software (Version 9.4, SAS Institute, North Carolina, United States).

### Ethics statement

This study was conducted in accordance with the French law. According to the French law, this non-interventional study based on anonymized data of authorized collections (declaration number CNIL: DE-2015-099, DE-2017-410) was not submitted to an ethics committee, and written parental consent was not needed.

## Results

### Population characteristics and exposure to drugs containing parabens

During the study period, the CPOE recorded 1,315 eligible neonates, and they represented our study population. The mean GA was 28.5 weeks, the mean birth weight was 1,129.9 g, and the sex ratio (M/F) was 1.06. Other characteristics of the study population are described in Table 2.

**TABLE 2:** Characteristics of the study population and exposure rate to drugs containing parabens.

	Study population N = 1,315
Birth weight (g), mean (±SD)	1,129.9 (±360.4)
Gestational age (weeks), mean (±SD)	28.5 (±2.1)
Male, n (%)	674 (51.3)
Year of admission, n (%)	
2017	228 (17.3)
2018	293 (22.3)
2019	244 (18.6)
2020	262 (19.9)
2021	288 (21.9)
Length of stay (days), median [min–max]	43.0 [1.0–199.0]
Mortality, n (%)	135 (10.3)
Exposure to drugs containing parabens, n (%)	1,124 (85.5)

Among the 1,315 neonates, 1,124 (85.5%) were exposed to medications containing parabens. There were key differences between exposed and unexposed infants, regarding sex, GA, birth weight, length of hospitalization, and mortality. The rate of exposure to drugs containing parabens was similar from 2017 to 2020 and significantly decreased in 2021. All these data are shown in Table 3.

**TABLE 3:** Comparison of infants exposed and not exposed to drugs containing parabens.

	Study population		
	Exposed *n* = 1,124	Not exposed *n* = 191	*p*-value
Birth weight (g), mean (±SD)	1,158.1 (±342.7)	963.0 (±414.3)	<0.0001^a^
Gestational age (weeks), mean (±SD)	28.7 (±2.0)	27.1 (±2.5)	<0.0001^a^
24–25	95 (57.6)	70 (42.4)	
26–27	219 (84.2)	41 (15.8)	
28–31	810 (91.0)	80 (9.0)	
Sex, n (%)			
Male	559 (82.9)	115 (17.1)	0.003^a^
Female	564 (88.7)	72 (11.3)	
Not determined at birth	1 (20.0)	4 (80.0)	
Year of admission, n (%)			
2017	200 (87.7)	28 (12.3)	0.01^b^
2018	251 (85.7)	42 (14.3)	
2019	216 (88.5)	28 (11.5)	
2020	232 (88.5)	30 (11.5)	
2021	225 (78.1)	63 (21.9)	
Length of stay (days), median [min–max]	48 [1–199]	7 [1–150]	<0.0001^a^
Mortality, n (%)	23 (17.0)	112 (83.0)	<0.0001^a^

^a^ χ^2^ test or Student’s t-test

^b^ Cochran–Armitage test for trend.

### Other exposure variables of interest

In exposed infants, the mean paraben intake was of 2.2 mg/kg/d, and the mean duration of exposure was of 33 days (Table 4). Infants born at lower GA received higher paraben intake and had longer exposure (*p* < 0.0001).

**TABLE 4:** Exposure to drugs containing parabens (PB) according to the gestational age.

		Gestational age (weeks of gestation)		
	Exposed infant *n* = 1,124	(24–25) *n* = 95	(26–27) *n* = 219	(28–31) *n* = 810
Daily PB intake, mg/kg/d				
Mean (±SD)	2.2 (1.4)	2.7 (1.2)	2.6 (1.4)	2.1 (1.4)
Median [p25%–p75%]	2.1 [1.0–3.1]	2.8 [1.8–3.8]	2.6 [1.6–3.3]	1.9 [0.9–2.9]
Min–max	0.1–11.1	0.1–5.2	0.2–11.1	0.2–10.8
Duration of PB exposure, days				
Mean (±SD)	33.1 (22.3)	50.4 (32.8)	45.3 (24.5)	27.8 (17.3)
Median [p25%–p75%]	30.0 [17.0–45.5]	53.0 [22.0–73.0]	47.0 [28.0–59.0]	27.0 [14.0–38.0]
Min–max	1.0–142.0	1.0–129.0	1.0–142.0	1.0–94.0
Cumulative PB intake, mg/kg				
Mean (±SD)	80.3 (84.6)	136.1 (104.3)	123.5 (103.1)	62.0 (67.8)
Median [p25%–p75%]	52.5 [20.5–116.0]	120.2 [52.7–212.7]	107.6 [50.1–167.5]	41.6 [17.4; 82.5]
Min–max	0.1–872.2	0.1–394.8	0.3–872.2	0.2–592.2
PB intake > 10 mg/k/d, n (%)	39 (3.5)	2 (2.1)	9 (4.1)	28 (3.5)
Duration of PB intake > 10 mg/k/d, days				
Mean (±SD)	6.3 (9.2)	1.5 (0.7)	9.2 (17.0)	5.8 (5.3)
Median [p25%–p75%]	3.0 [2.0–7.0]	1.5 [1.0–2.0]	2.0 [1.0–5.0]	3.5 [2.5–7.5]
Min–max	1.0–53.0	1.0–2.0	1.0–53.0	1.0–23.0
DOL at the first exposure				
Median [min–max]	14 [1–183]	25 [6–162]	18 [4–183]	13 [1–135]
DOL 1–7, n (%)	117 (10.3)	2	9	108
DOL 8–14, n (%)	455 (40.4)	17	68	372
DOL 15–21, n (%)	270 (23.9)	27	56	189
DOL 22–28, n (%)	82 (7.2)	12	21	51
DOL >28, n (%)	205 (18.1)	42	70	95
DOL at the last exposure				
Median [min–max]	53 [5–199]	105 [14–180]	75 [13–199]	46 [5–159]
DOL 1–7, n (%)	3 (0.2)	1	1	3
DOL 8–14, n (%)	33 (2.8)	2	3	30
DOL 15–21, n (%)	76 (6.7)	3	3	72
DOL 22–28, n (%)	74 (6.5)	3	2	71
DOL >28, n (%)	943 (83.8)	91	215	639

DOL, day of life; PBs, parabens.

The exposure to drugs containing parabens occurred more often during the second week of life, and it occurred previously for infants with higher GA at birth. Among the exposed infants, 39 (3.5%) received paraben intake exceeding the ADI. The number of days with paraben intake superior to the ADI ranged from 1 to 53. The exposure rate was significantly higher in female infants compared to male infants. Other exposure variables of interest are summarized in Table 4.

### Drugs containing parabens

**FIGURE 1:**
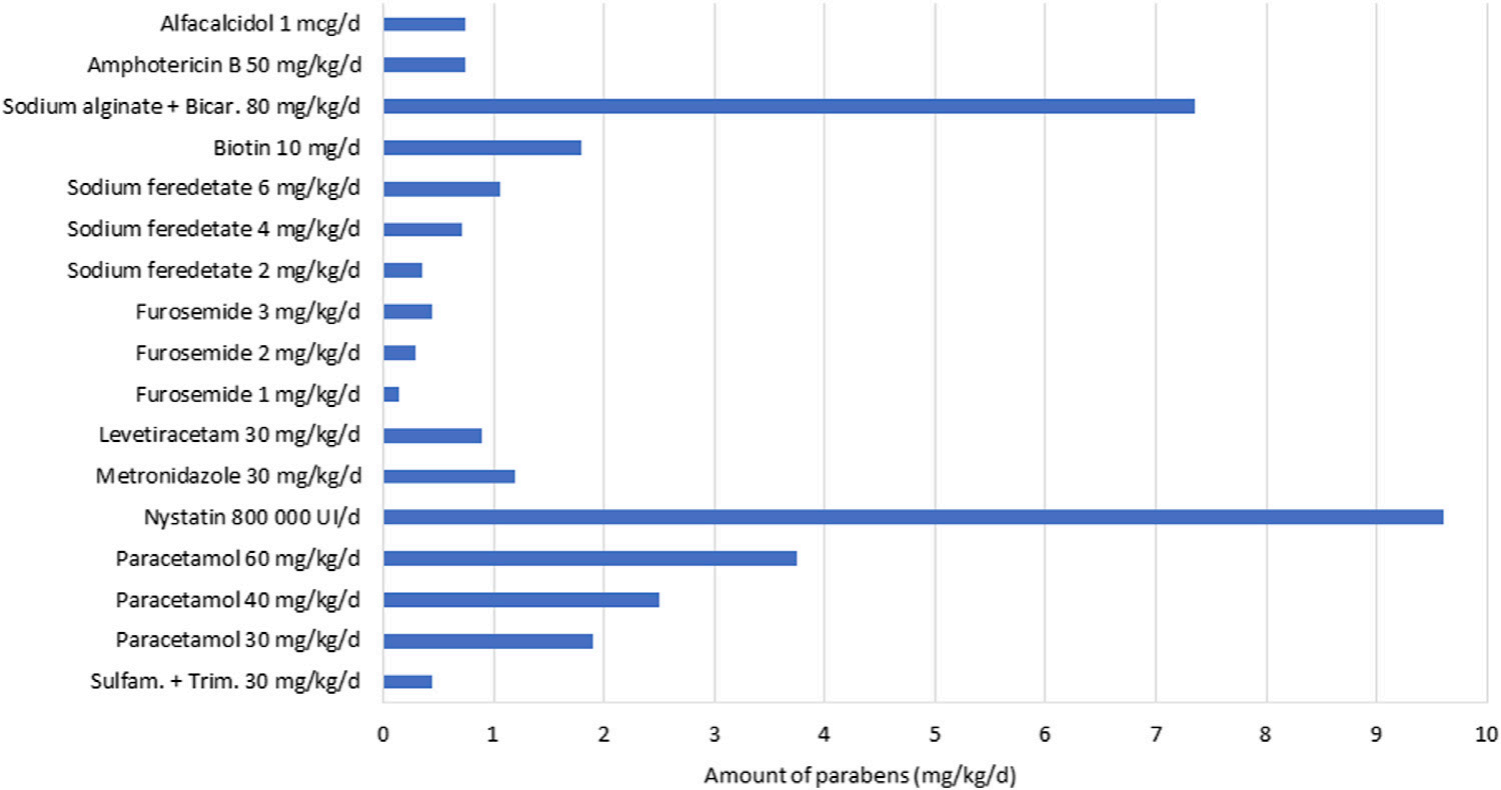
Daily paraben amount according to drug dosage.

The top five administered drugs (international non-proprietary name, INN) containing parabens were: sodium ferrous feredetate, paracetamol (acetaminophen), furosemide, sodium bicarbonate + sodium alginate, and amphotericin B. Table 5 shows the exposure rate for each paraben-containing drug and the paraben amount provided by the top five drugs. The number of drugs containing parabens in exposed infants ranged from 1 to 5. Most of the exposed patients (54.8%) were exposed to two different drugs containing parabens. Among the 1,124 exposed infants, 251 (22.3%), 207 (18.4%), 47 (4.2%), and 3 (0.3%) received 1, 3, 4, and 5 drugs, respectively (data not shown). Figure 1 illustrates the daily amount of parabens according to drug dosage, for 10 drugs containing parabens.

**TABLE 5:** Exposure rate to each paraben-containing drug and cumulative intake of parabens via the top five paraben-containing drugs.

	Exposed infants *n* = 1,124
Exposure to paraben-containing drug, n (%)	
Sodium feredetate	1,064 (94.7)
Paracetamol	847 (75.4)
Furosemide	175 (15.6)
Sodium alginate + sodium bicarbonate	124 (11.0)
Amphotericin B	39 (3.5)
Josamycin	29 (2.6)
Nystatin	8 (0.7)
Alfacalcidol	7 (0.6)
Levetiracetam	5 (0.4)
Rifampicin	4 (0.4)
Biotin	1 (0.1)
Domperidone	1 (0.1)
Lamivudine	1 (0.1)
Metronidazole	1 (0.1)
Sulfamethoxazole + trimethoprim	1 (0.1)
Cumulative PB intake, mg/kg, median [min–max]	
Sodium feredetate	19.8 [0.4–106.9]
Paracetamol	42.5 [1.9–431.3]
Furosemide	0.3 [0.0–17.3]
Sodium alginate + sodium bicarbonate	73.6 [7.4–552.0]
Amphotericin B	7.5 [0.8–21.0]

For four drugs, the usually administered posology exceeded 2 mg/kg/d of parabens: sodium bicarbonate + sodium alginate at 80 mg/kg/d, nystatin at 800,000 IU/d, paracetamol at 40 mg/kg/d, and paracetamol at 60 mg/kg/d.

## Discussion

This study aimed to quantify the exposure to parabens through drugs administered to VPIs cared for in the NICU. Our results show that most of this vulnerable population was exposed to paraben-containing medications during the first hospitalization, from birth until discharge. Exposed infants, compared to the unexposed ones, had more often a higher birth weight and GA and were more often girls. The analysis of a large cohort, with prospective and comprehensive data collection over 5 years, is an important achievement that distinguishes our study.

A monocentric prospective study performed by Binson et al. (2020) quantified the exposure to parabens in 22 neonates, born at term or preterm, and hospitalized over a one-month period. The authors showed that preterm infants received a cumulative dose of parabens (MP + EP + PP) equal to 10.9 mg/kg. Our results showed a cumulative dose of 80.3 mg/kg. These data are difficult to compare because of the length of hospital stay, and therefore the duration of exposure was very different in the two studies.

In our cohort, the mean daily amount of MP + EP was higher than that of the study by Binson et al. (2020), in which the mean daily amount of MP and EP in 17 preterm infants was, respectively, 0.41 and 0.30 mg/kg. This could be explained by the greater size of our population. Moreover, a substantial number of extremely preterm infants were included in the present study, and this population is particularly exposed to drugs during the stay in the NICU.

Among the exposed infants of our cohort, 3.5% of babies received a daily intake exceeding the ADI of 10 mg/kg/d for the sum of MP and EP, established by the EFSA (EFSA, 2004). The duration of exposure to paraben amounts higher than the ADI could be as longer as 53 days. Based on our results, we can conclude that VPIs receiving multiple medications in the NICU can potentially achieve such amounts.

In terms of frequency of exposure, the four most used preparations were oral solutions or suspensions: Ferrostrane^®^ 0.68% (sodium feredetate), Doliprane^®^ 2.4% (paracetamol), Lasilix^®^ 10 mg/mL (furosemide), and Gaviscon^®^ 10 mL (sodium bicarbonate + sodium alginate). It is interesting to note that all these medications are approved for use in neonates, including preterm infants. The French (European) Summary of Product Characteristics for licensed medications (Agence-prd, 2005) approves these drugs in terms of formulation, indication, and dosage, and these compounds are very often used in French NICUs. A multicenter French study on 27,382 neonates cared for in NICUs over a two-year period showed that 12 INNs were prescribed for at least 10% of patients. Among them, paracetamol was administered to 36.9% and oral iron to 24% of infants during the hospital stay (Gouyon et al., 2019).

Because of their reduced body store at birth and their higher demand during catch-up growth, all low birth weight infants should receive an iron supplement to prevent iron deficiency anemia (Mills and Davies, 2012). In VPIs, iron supplementation may have long-lasting effects on behavioral functions (Berglund et al., 2018). The ESPGHAN Committee on nutrition recommends a daily iron intake of 2–3 mg/kg/d starting at 2 weeks of age for very low birth weight infants and states that infants who receive erythropoietin treatment may need a higher dose (up to 6 mg/kg/d) (Embleton et al., 2023). In clinical practice, enteral iron administration is started as early as enteral feeding is tolerated and then is progressively increased (Lapillonne and Becquet, 2017). Paracetamol, furosemide, and sodium bicarbonate + sodium alginate are used for the management of very common conditions or symptoms in preterm infants (pain, salt and fluid overload, and gastroesophageal reflux, respectively). Interestingly, our study showed that when sodium bicarbonate + sodium alginate is administered at the usual posology of 80 mg/kg/d, the amount of parabens achieves 7.36 mg/kg/d. Thus, if the infant receives this medication in combination with paracetamol at 40 mg/kg/d and sodium feredetate at 6 mg/kg/d, paraben intake will exceed the ADI. These results led us to search for alternative paraben-free pharmaceutical formulations. Thus, we identified for both iron and paracetamol, two available paraben-free oral formulations (iron AP-HP-newborn^®^ capsules, 0.5 mg and EFFERALGANMED^®^ suspension, 30 mg/mL, respectively). These formulations are currently under consideration by the local hospital pharmacy for in-hospital administration in newborn infants. Our results documented significant differences between exposed and unexposed infants. The shorter length of stay of unexposed patients can be explained by the fact that this group had a higher mortality rate. The time-point of exposure occurred later for premature infants born at 24–25 weeks of gestation, as paraben-containing medications mentioned previously are mostly given by the oral route. This route of administration (that generally starts during the second week of life) is often delayed in extremely preterm infants (born at less than 28 weeks of gestation) due to enteral immaturity, clinical instability, and a longer critical phase after birth.

Exposure to parabens may also occur through various sources, other than drugs, in the NICU. An exploratory study (Iribarne-Durán et al., 2019) showed that a large variety of medical products are potential sources of exposure to parabens with *in vitro* endocrine activity: plastic stopcocks, transparent film dressings, enteral feeding tubes, sterile gloves, umbilical catheters, umbilical or intravenous catheters, intravenous infusion extension kits, and protective goggles for phototherapy. Recently, a multicenter point-prevalence study was conducted in Japan in order to investigate neonatal exposure to “potentially harmful excipients” (Saito et al., 2021). This study included 343 newborn infants from 22 NICUs and showed that enema formulations accounted for the highest proportion (45.8%) of paraben-containing prescriptions. Parabens were the most common excipient in topical prescriptions as they were present in one topical product administered to 14.3% of included neonates. Finally, in relation to the issue of potential sources of endocrine disruptors, it has also been shown that dietary habits (consumption of bakery or packaged products) and cosmetics use had a positive association with urinary levels of parabens in breastfeeding mothers (Sanchis et al., 2020). These results emphasize the need to adopt preventive strategies in order to minimize sources of exposure for these vulnerable patients.

An interesting finding of our study was that paraben exposure was significantly higher in female than male infants. Fundamental and clinical studies (Bräuner et al., 2023) showed that paraben exposure may have sex-specific effects during perinatal life, and thus this difference deserves further investigation in future studies on VPIs.

Our study has several limitations. First, exposure—as specifically defined in clinical pharmacology and related to pharmacokinetics—is not available in this dataset. Studies on human subjects described metabolic and excretion processes of parabens after oral ingestion (Guo et al., 2017; Sakhi et al., 2018). Following excretion, the parent compounds can be measured in urine and have been shown to be valid biomarkers of exposure. Animal studies revealed that absorption is complete after intravenous injection, with no tissue accumulation and rapid urine excretion (No, 2008). Our study could not assess the metabolic consequences of paraben intake or whether differences exist according to the administration route. Second, we did not consider other potential sources of exposure to parabens. Another limitation is the lack of a greater number of clinical covariates and outcomes in our cohort. This information would have been interesting to know if other characteristics were associated with an increased risk of exposure.

Our study has several strengths. It exhaustively reflects drug administration in a homogeneous group of vulnerable infants. Data collection is prospective, rigorous, and corresponding to real prescriptions by a certified CPOE approved by the French National Authority for Health (HAS). In addition, the quantification of paraben amount by drugs is accurate, thanks to the Thériaque^®^ database. Finally, this regional-based study is aligned with the national and regional health strategies that aim to identify and reduce the environmental exposure to endocrine disruptors, during the pivotal period of the first “1,000 days” (Ministère des Solidarités et de la Santé, 2019).

In conclusion, commonly used drugs are potential sources of paraben exposure in neonates. The ADI of 10 mg/kg/d was occasionally exceeded in VPIs cared for in the NICUs in this regional setting. Premature infants, and even more so, extremely premature infants, are fragile neonates, and environment plays a significant impact on their development. An important challenge, on a regional basis, will be to explore the availability of alternative pharmaceutical preparations containing fewer parabens or being paraben-free. More broadly, further studies are warranted to quantify overall, prolonged, and cumulative exposure to parabens during this critical period for lifelong health.

## Data Availability

The raw data supporting the conclusion of this article will be made available by the authors, without undue reservation.

## Abbreviations

ADI, acceptable daily intake; BP, butylparaben; CPOE, computerized prescriber order entry; DOL, day of life; EFSA, European Food Safety Authority; EP, ethylparaben; GA, gestational age; INN, International Non-proprietary Name; MP, methylparaben; NICU, neonatal intensive care unit; PP, propylparaben; VPIs, very preterm infants.
